# Outcomes of non-infectious Paediatric uveitis in the era of biologic therapy

**DOI:** 10.1186/s12969-018-0266-5

**Published:** 2018-08-06

**Authors:** Megan Cann, Athimalaipet V. Ramanan, Andrew Crawford, Andrew D. Dick, Sarah L. N. Clarke, Fatima Rashed, Catherine M. Guly

**Affiliations:** 1 University Hospitals Bristol NHS Foundation Trust, Bristol, UK; 2 Translational Health Sciences, Bristol Medical School, Faculty of Health sciences, Bristol, UK; 3 BHF Centre for Cardiovascular Science, Queen’s Medical Research Institute, University of Edinburgh, Edinburgh, UK; 4 MRC Integrative Epidemiology Unit, Population Health Sciences, University of Bristol, Bristol, UK; 5 National Institute for Health Research (NIHR) Biomedical Research Centre at Moorfield Eye Hospital, London, UK; 6 University College London Institute of Ophthalmology, London, UK

**Keywords:** Uveitis, Biologics, Paediatrics, Visual outcomes

## Abstract

**Background:**

There is a paucity of data on the ocular outcomes in paediatric non-infectious uveitis since the introduction of the biologic agents. The purpose of this study was to outline the clinical characteristics of children with non-infectious uveitis and determine the visual outcomes and ocular complication rates in the modern era.

**Methods:**

Children with non-infectious uveitis from January 2011 to December 2015 were identified. Data was collected at baseline, 1, 3, 5, and 10 years post diagnosis. The incidence rates of visual impairment, structural ocular complications and surgical intervention were calculated. Using logistic regression the association between various baseline characteristics and later visual impairment was investigated.

**Results:**

Of the 166 children, 60.2% (*n* = 100) had a systemic disease association. 72.9% (*n* = 121) children received methotrexate, 58 children progressed to a biologic. The incidence rates of visual acuity loss to > 0.3 LogMAR (6/12) and to ≥1.0 LogMAR (6/60) were 0.05/Eye Year (EY) and 0.01/EY, respectively. Visual outcomes in the Juvenile Idiopathic Arthritis associated Uveitis (JIA-U) and Idiopathic Uveitis cohorts were not statistically significant. Of the 293 affected eyes, posterior synechiae was the predominant complication on presentation, while cataract had the highest incidence rate (0.05/EY). On direct comparison, children with JIA-U were statistically significantly more likely to develop glaucoma while children with Idiopathic Uveitis were statistically significantly more likely to develop macular oedema.

**Conclusion:**

One third of children received a biological therapy, reflecting increasing utilisation and importance of biological agents in the management of inflammatory conditions. Rates of visual impairment and ocular complications are an improvement on previously published data.

## Background

Uveitis is rare in the paediatric population, with an estimated incidence of 4.3 per 100,000 and a prevalence of 27.9 per 100,000 [1, 2] but there is a high rate of chronic disease [3]. Non-infectious uveitis accounts for between 69 and 95% of childhood uveitis [4–7]. Juvenile Idiopathic Arthritis associated Uveitis (JIA-U) makes up 41–47% of cases but equally in 28–51% of children no cause is found [7–9]. Ocular complications including cataract, glaucoma and macular oedema are reported in up to 76% of all cases of paediatric uveitis [4]. Visual impairment is common but reporting is variable making comparisons difficult. Using incidence rates of visual impairment, visual loss in children with JIA-U has been reported at 0.10/EY [10]. Other studies have reported overall rates of visual impairment in at least one affected eye between 17 and 37% and 5 year rates of 36.36 and 15.1% for visual acuity worse then 20/50 and 20/200 respectively [3, 4, 8].

Topical corticosteroids carry a risk of cataract and glaucoma [11] and increasingly early use of immunosuppressive agents is advocated in chronic non-infectious uveitis to reduce the risk of visual loss [12, 13]. Methotrexate is the most commonly prescribed immunosuppressive therapy in paediatric uveitis [13], but 27–48% children do not achieve control of inflammation and 20% experience adverse events [14–16]. Use of infliximab in the management of refractory paediatric uveitis was first reported in 2005 [17]. The following year a small case series of adalimumab for paediatric uveitis was published [18]. Adalimumab was licenced for adult and paediatric non-infectious uveitis in 2016 and 2017 respectively following successful outcome of randomised controlled trials (RCTs) [19, 20].

There is a paucity of data on longer term outcomes of paediatric uveitis (including JIA-U) in the era of biologic treatment. The aim of this retrospective study is to determine the visual outcomes and ocular complications of children attending a tertiary service covering the South-West of England and the South of Wales (approximate population of 5.5 million).

## Methods

### Patient identification

Bristol Eye Hospital Databases were reviewed to identify all patients with uveitis. Children were included if they had been diagnosed with non-infectious uveitis prior to the age of 16 years and been managed at the Bristol Eye Hospital between January 2011and December 2015. Children referred to the service were either resident in Bristol and referred from local medical services (primary referrals) or lived outside Bristol and were referred from surrounding ophthalmology services(tertiary referrals).

### Data collection

Data was collected retrospectively at standard time intervals; diagnosis, and 1, 3, 5 and 10 years. The appointment closest to the time interval was selected for analysis. Demographic data included age at diagnosis, gender and ethnicity. Clinical uveitis details included aetiology, laterality, anatomical location using Standardization of Uveitis Nomenclature (SUN) criteria [21] and structural complications. Elevated intraocular pressure (IOP) was defined as > 21 mmHg and hypotony as < 5 mmHg. Pharmacological and surgical treatments were documented. Visual acuity was recorded in logMAR. LogMAR is the logarithmic representation of visual acuity. It is the accepted standard of representing visual acuity in research as it is recognised to be more reliable, discriminative and repeatable when compared to the classic Snellen’s chart [22]. Visual impairment was stratified by severity as logMAR > 0.3 (worse than 6/12) or ≥ 1.0 (6/60 or worse) [21].

### Statistical analysis

The incidence rates of visual impairment and structural ocular complications were calculated for the whole population, and for the JIA-U and idiopathic uveitis cohorts. Not all patients contributed at all time points. Incidence rates were calculated per at risk eye year using longitudinal data analysis to account for variable length of follow up [23]. Year of complication onset was not recorded for 20 out of a total of 247 events (band keratopathy *n* = 5, cataracts *n* = 8, glaucoma *n* = 3, posterior synechiae *n* = 1, elevated IOP *n* = 3). A year of onset of 4 years was used for these events as this was the nearest whole year to the study cohort’s mean years of follow-up (range from 3.8 to 4.5 years). The association between various baseline characteristics and later visual impairment was investigated using logistic regression (crude and adjusting for age and sex). Analyses allowed for intragroup correlation between eyes in patients with bilateral events. Analyses were performed using Stata 14 statistical software (Stata Corp, College Station, Texas).

Data collection was approved by the hospital trust and ethics committee approval was not required.

## Results

**Fig. 1:**
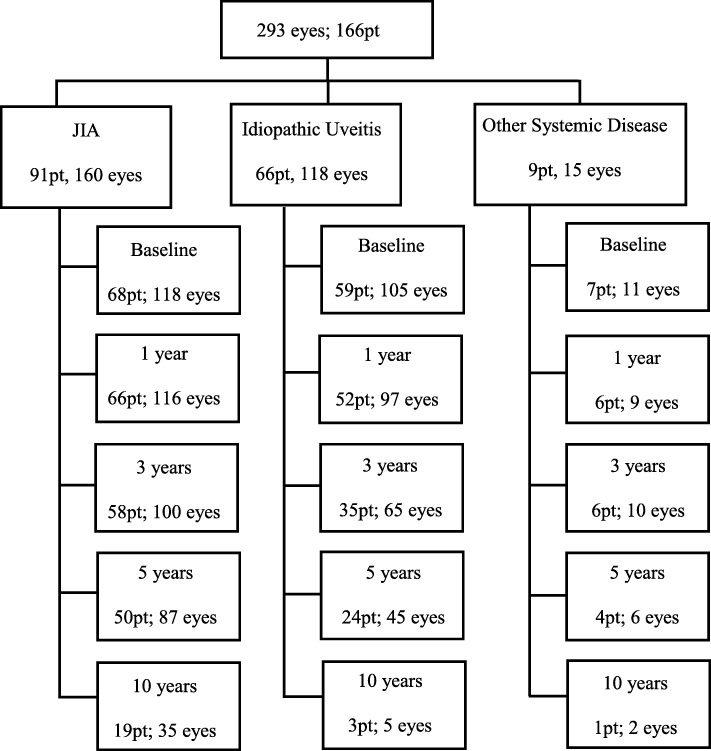
Data Available at Standard Time Intervals Detailed information of the number of patients and affected eyes per aetiology at baseline, 1 year, 3 years, 5 years and 10 years post diagnosis

Patient demographics and clinical characteristics of the 166 patients included in the study are outlined in Table 1. Of the 50 children with unilateral uveitis on presentation, 11 progressed to bilateral disease. A total of 293 affected eyes were included in the study. Baseline information was available for 234 eyes in total. 60.2% of patients had an underlying systemic disease association (Fig. 1). JIA was the primary associated systemic disease, however Tubulointerstitial Nephritis and Uveitis (TINU; 4 patients, 6 eyes), Blau (3 patients; 6 eyes), Behcet (1 patient; 1 eye) and undifferentiated inflammatory disease (1 patient; 2 eyes) was also present. Anterior uveitis was the most common form of uveitis occurring in 125 patients (75.3%). In JIA 89 out of 91 patients (97.8%) had anterior uveitis. Unfortunately time to diagnosis data was not available for the cohort. Of the 93 tertiary referral patients (i.e. referred from surrounding ophthalmology services for subspecialist opinion); 64.5, 8.6, 10.8, 6.5 and 9.7% were seen at the tertiary centre within 3 months, 1 year, 3 years, 5 years and 10 years of diagnosis respectively.

**Table 1:** Patient Demographic and Clinical Characteristics

	Cohort (%)	JIA (%)	Other Associated Systemic Disease(%)	Idiopathic (%)
No. of Patients	166	91 (54.8)	9 (5.4)	66 (39.8)
Female	99 (59.6)	62 (68.1)	2 (22.2)	35 (53)
Age at diagnosis				
Average	8.03y	5.9y	10.5y	10.7y
Median	7y	5y	12y	11y
Referral type				
Tertiary	93 (56)	56 (61.5)	6 (66.7)	31 (47)
Ethnicity				
Caucasian	128 (77.1)	73(80.2)	6 (66.7)	49 (74.2)
Asian	8 (4.8)	2 (2.2)	2 (22.2)	4 (6)
African	1 (0.6)	0	0	1 (1.5)
Other	7 (4.2)	2 (2.2)	1 (11.1)	4 (6)
Unknown	22 (13.3)	14 (15.4)	0	8 (12.1)
Laterality on presentation				
Bilateral	116 (69.9)	62 (68.1)	5 (55.6)	49 (74.2)
Anatomic Localisation				
Anterior	125 (75.3)	89 (97.8)	5 (55.6)	31 (47)
Intermediate	29 (17.5)	0	1 (11.1)	28 (42.4)
Posterior	1 (0.6)	0	0	1 (1.5)
Panuveitis	11 (6.6)	2 (2.2)	3 (33.3)	6 (9.1)
Visual Impairment at baseline (per eye)				
> 0.3logMAR	43 (18.4)	22 (18.6)	3 (27.3)	18 (17.1)
≥ 1.0 logMAR	10 (4.3)	7 (5.9)	0	3 (2.9)
Medication use over study				
Corticosteroids				
Topical CS	155 (93.4)	91 (100)	8 (88.9)	56 (84.8)
Systemic CS	58 (34.9)	28 (30.8)	7 (77.8)	23 (34.8)
Peri/Intraocular	12 (7.2)	6 (6.6)	3 (33.3)	6 (9.1)
Steroids				
Conventional DMARD	121 (72.9)	82 (90.1)	6 (66.7)	33 (50)
Methotrexate	121 (72.9)	82 (90.1)	6 (66.7)	33 (50)
Only Methotrexate	47 (28.3%)	35 (38.5)	1 (11.1)	11 (16.7)
Mycophenolate	36 (21.7)	19 (20.9)	2 (22.2)	15 (22.7)
mofetil				
Tacrolimus	6 (3.6)	3 (3.3)	0	3 (4.5)
Ciclosporin	3 (1.8)	2 (2.2)	0	1 (1.5)
Biologics	58 (34.9)	43 (47.3)	5 (55.6)	10 (15.2)
Adalimumab	52 (31.3)	38 (41.8)	5 (55.6)	9 (13.6)
Infliximab	19 (11.4)	14 (15.4)	4 (44.4)	1 (1.5)
Abatacept	3 (1.8)	3 (1.8)	0	0
Tocilizumab	2 (1.2)	2 (1.2)	0	0

At presentation 118 eyes JIA, 11 other associated systemic diseases and 105 idiopathic

Medications are recorded in Table 1. The first line systemic immunosuppressive agent for all children was methotrexate. Of the 166 patients, 39 (23.5%) children received additional conventional DMARD therapy; either as monotherapy or in combination with Methotrexate (20/91 JIA patients, 17/66 idiopathic uveitis patients and 2/9 patients with other systemic diseases). 58 children (34.4%) received a biologic agent and 14 children (8.4%) required ≥2 biologics over the follow up period. No child received a biologic agent at baseline. Biologics were more likely to be delivered to children with JIA-U (43 children; 47.3%) than those with idiopathic uveitis (10 children; 15.2%). 7 (10.6%) children with idiopathic uveitis did not require treatment (*p* = < 0.001).

A total of 678 person years and 1216 eye years (EY) of follow up were available. For the total population the median duration of follow up was 5 years. Children with JIA-U had a longer duration of follow up when compared to children with idiopathic uveitis (median 5 years vs 3 years). Availability of follow up data is outlined in Fig. 1. 34 children contributed only 1 visit; the majority of these children (21 children) were tertiary referrals seen at least 1 year post diagnosis. The median number of evaluation visits per patient was 3. Rates of complications at presentation and incidence rates of newly diagnosed complications are summarised in Table 2. The rates of visual acuity loss over the period of observation to > 0.3 LogMAR and to ≥1.0 LogMAR among affected eyes were 0.05/EY and 0.01/EY, respectively. At presentation 81/234 eyes (34.6%) had at least one ocular uveitis associated complication, of which posterior synechiae was the most common (43 eyes; 18.4%). The rate of newly diagnosed band keratopathy and posterior synchiae was 0.02/EY. Children with JIA-U were more likely to develop raised IOP (*p* = 0.05) and glaucoma (*p* = 0.002) when compared to children with idiopathic uveitis, while children with idiopathic uveitis were more likely to developed macular oedema (*p* = 0.01) over the period of follow up. Cataract was the frequent complication to occur over the follow up period with an incidence rate of 0.05/EY.Three eyes (3 children) had amblyopia. All three eyes had visual impairment, 2 eyes had ≥1.0 logMAR and one had > 0.3logMAR. There was no statistical difference between the rate of visual impairment between children with anterior uveitis versus children with intermediate uveitis. The incidence rate of visual impairment > 0.3 logMAR for anterior uveitis was 0.04/EY (CI95% 0.03–0.06) compared to 0.05/EY (CI95% 0.02–0.11) for children with intermediate uveitis (*p* = 0.80). No child with intermediate uveitis developed a visual impairment of ≥1.0logMAR over the study period whereas the incidence in children with anterior uveitis was 0.01/EY (CI95% 0.01–0.02) (*p* = 0.13). Additionally, there was no significant difference between rates of visual impairment in those children with JIA-U anterior uveitis and non-JIA-U anterior uveitis. Children with JIA-U anterior uveitis had an incidence rate of visual impairment > 0.3logMAR of 0.05/EY (CI95% 0.03–0.07) compared to 0.02/EY (CI95% 0.01–0.07) in the non-JIA-U anterior group (*p* = 0.19). The rate of visual impairment ≥1.0logMAR between the JIA-U anterior uveitis and the non-JIA-U anterior uveitis cohort was 0.01/EY (CI95% 0.00–0.05) and 0.01/EY (CI95% 0.01–0.03) respectively (*p* = 0.5).

**Table 2:** Complication Incidence Rates

	Total Cohort			JIA-Uveitis			Idiopathic Uveitis			
	Eyes On Presentation *n* = 234^ (%)	n/EY*	Incidence Rate/EY (CI 95%)	Eyes On Presentation *n* = 118^ (%)	n/EY	Incidence Rate/EY (CI 95%)	Eyes On Presentation *n* = 105^ (%)	n/EY	Incidence Rate/EY (CI 95%)	IU vs JIA-U Incidence *p* = 0.05
Visual Impairment										
> 0.3 LogMAR	43 (18.4%)	39/830	0.05 (0.03–0.06)	22 (18.6%)	27/529	0.05 (0.04–0.07)	18 (17.1%)	11/260	0.04 (0.02–0.08)	0.69
≥ 1.0 LogMAR	10 (4.3%)	14/1034	0.01 (0.01–0.02)	7 (5.9%)	10/652	0.02 (0.01–0.03)	3 (2.9%)	2/335	0.01 (0.00–0.02)	0.15
Ocular Complication										
Cataract	17 (7.3%)	47/939	0.05 (0.04–0.07)	11 (9.3%)	31/578	0.05 (0.04–0.08)	5 (4.8%)	12/317	0.04 (0.02–0.07)	0.24
Raised IOP	19 (8.1)	22/986	0.02 (0.02–0.04)	13 (11%)	17/606	0.03 (0.02–0.05)	6 (5.7%)	3/327	0.01 (0.00–0.03)	**0.05**
Glaucoma	0	15/1062	0.01 (0.01–0.02)	0	15/657	0.02 (0.01–0.04)	0	0/352		**0.002**
Posterior Synechiae	43 (18.4%)	21/860	0.02 (0.02–0.04)	23 (19.5%)	17/524	0.03 (0.02–0.05)	18 (17.1%)	4/296	0.01 (0.01–0.04)	0.14
Macular Oedema	7 (3%)	14/1027	0.01 (0.01–0.02)	3 (2.5%)	4/681	0.01 (0.00–0.02)	4 (3.8%)	9/293	0.02 (0.02–0.06)	**0.01**
Band Keratopathy	19 (8.1%)	20/938	0.02 (0.01–0.03)	15 (12.7%)	12/586	0.02 (0.01–0.04)	3 (2.9%)	6/314	0.02 (0.01–0.04)	0.77
Hypotony	4 (1.7%)	3/1084	0.00 (0.00–0.01)	3 (2.5%)	2/686	0.00 (0.00–0.01)	1 (1%)	0/347		0.31
Optic Disc Swelling	23 (9.8%)	3/1012	0.00 (0.00–0.01)	2 (1.7%)	2/690	0.00 (0.00–0.01)	19 (18.1%)	1/279	0.00 (0.00–0.03)	0.81
Vitreous Haemorrhage	0	1/1108	0.00 (0.00–0.01)	0	1/703	0.00 (0.00–0.01)	0	0/352		0.68
Epiretinal Membrane	1 (0.4%)	1/1107	0.00 (0.00–0.01)	0	0/707	0.00	1 (1%)	0/349		0.68
Surgical Procedure										
Cataract Removal	2 (0.9%)	22/1038	0.02 (0.01–0.03)	2 (1.7%)	18/639	0.03 (0.02–0.04)	0	3/350	0.01 (0.00–0.03)	**0.04**
Trabeculectomy	0	11/1087	0.01 (0.01–0.02)	0	11/682	0.02 (0.01–0.03)	0	0/352		**0.01**
Vitrectomy	2 (0.9%)	8/1074	0.01 (0.00–0.01)	2 (1.7%)	4/673	0.01 (0.00–0.02)	0	4/348	0.01 (0.00–0.03)	0.32
Laser Capsulotomy	0	3/1112	0.00 (0.00–0.01)	0	3/707	0.00 (0.00–0.01)	0	0/352		0.32
Iridectomy	0	2/1107	0.00 (0.00–0.01)	0	2/702	0.00 (0.00–0.01)	0	0/352		0.47

*n/EY – Number of new events/Number of Eye Years of Follow up

^ Within the cohort 293 eyes were affected. However, not all children had baseline presentation data available

Surgical procedures were required in 38 eyes and in some cases one eye received multiple procedures. Cataract extraction was the most common procedure, performed in 24 eyes (0.9% within 3 months of presentation; Incidence rate 0.02/EY). 20 eyes received an intraocular lens implant and 3 remained aphakic. Lens insertion data was not available for 1 eye. Trabeculectomy was required in 11 eyes (0.01/EY) and vitrectomy in 10 eyes (0.9% within 3 months of presentation; 0.01/EY). Indication for vitrectomy is as follows; uncontrolled inflammation despite aggressive therapy (3 eyes), vitreal debris (3 eyes), cyclitic membrane (1 eye) and undocumented reasons (3 eyes) Patients with JIA were more likely to require cataract removal and trabeculectomy than those with idiopathic uveitis (*p* = 0.04 and *p* = 0.01 respectively) over the period of follow up. Two children required an enucleation at 5 and 10 years follow up. Both eyes had severe sight loss and required removal due to chronic pain.

Using logistic regression, demographic and clinical characteristics on presentation were analysed to determine whether they were predictive of visual impairment. These characteristics are detailed in Table 3. Tertiary referral patients were statistically more likely to develop a visual impairment of logMAR > 0.3 (*p* = 0.03) and ≥ 1.0 (*p* = 0.05). The presence of posterior synechiae at presentation was also a risk factor for visual impairment ≥1.0 logMAR (*p* = 0.01).

**Table 3:** Patient Risk Factors at Baseline for Visual Impairment

	Risk of Visual Impairment > 0.3logMAR		Risk of Visual Impairment ≥1.0 logMAR	
	OR (95%CI)	p (0.05)		p (0.05)
Demographics				
Age at uveitis diagnosis	0.97 (0.87–1.08)	0.56	1.02 (0.88–1.19)	0.76
Bilateral Disease	1.23 (0.46–3.34)	0.68	0.45 (0.12–1.65)	0.23
Female	1.43 (0.61–3.36)	0.41	1.24 (0.38–4.12)	0.72
Tertiary Referral	2.71 (1.12–6.56)	0.03	4.59 (0.98–21.47)	0.05
Aetiology				
JIA	2.05 (0.88–4.77)	0.10	2.15 (0.62–7.43)	0.23
Idiopathic	0.54 (0.23–1.29)	0.16	0.23 (0.05–1.09)	0.07
Uveitis Characteristics				
Anterior Uveitis	0.89 (0.37–2.14)	0.80	0.89 (0.26–3.07)	0.85
Bilateral Disease	1.23 (0.46–3.34)	0.68	0.45 (0.12–1.65)	0.23
Complications at Baseline				
Band keratopathy	1.24 (0.24–6.37)	0.80	1.12 (0.15–8.53)	0.92
Cataracts	1.43 (0.29–6.99)	0.66	5.16 (0.91–29.23)	0.06
Posterior synechiae	0.84 (0.26–2.71)	0.77	4.91 (1.38–17.39)	0.01
Elevated IOP	0.35 (0.05–2.51)	0.29	1.00	
Macular oedema	1.09 (0.12–10.07)	0.94	1.00	
Hypotony	2.20 (0.19–25.31)	0.53	7.08 (0.64–78.00)	0.11
Optic disc swelling	0.98 (0.29–3.30)	0.97	2.05 (0.47–8.88)	0.34

## Discussion

We report a large cohort of children with non-infectious uveitis managed in a UK tertiary unit. Use of systemic immunosuppression was high with 72.9% of children receiving methotrexate and 34.9% receiving a biologic agent. The recent publication of the SYCAMORE trial [20] has supported the efficacy of rapid escalation to biologics in the management of children with JIA-U refractory to conventional immunosuppressive therapy.

Despite published evidence that up to 73% of children experience improvement in intraocular inflammation with methotrexate [14], in this population methotrexate refractory disease was common. Of the 121 children started on methotrexate, 58 (47.9%) required third line therapy in the form of one or more biologic agent. Tertiary referral patients were 1.6 times more likely to be treated with a biologic when compared to primary referral patients (41.9% vs 26%). This difference was statistically significant (*p* = 0.03). Children with an associated systemic disease were statistically more likely to receive a biologic than those with idiopathic uveitis (48% vs 15.2% *p* = < 0.001). However the rates of visual impairment were similar in the JIA-U and idiopathic uveitis cohorts and so the difference in treatment may represent a milder disease course of idiopathic uveitis or the longer median duration of follow up of the JIA-U cohort. Overall rates of biologic use are higher than previous reports. The rate of biologic use in the idiopathic uveitis cohort (15.2%) is slightly lower than reported in Sardar et al. [24] where 21% of patients required third line therapy. However, the proportion of children with idiopathic panuveitis and posterior uveitis was slightly higher in the French cohort, which may explain the slightly higher rate of biologic use. In 2007 Saurenmann et al. [25] reported that 11% of children with JIA-U received an anti-TNF agent but over the last 10 years biologics have become more widely available and accepted practice for refractory JIA-U.

Visual outcomes in this cohort are an improvement on previously published data. Thorne et al. [10] reported in a population of children with JIA-U rates of visual impairment of 6/15 or worse (≥0.4 logMAR) and 6/60 or worse (≥1.0 logMAR) of 0.10/UEY and 0.08/EY respectively. In our study, the incidence rate of visual impairment in those children with JIA-U was 0.05/EY (> 0.3logMAR) and 0.02/EY (≥1.0logMAR). While JIA-U had a higher incidence rate of visual impairment compared to idiopathic uveitis, the difference was not significant. Comparison between rates of ocular complications within our study population and previous published data is outlined in Table 4. In this study eight children developed bilateral visual impairment > 0.3 logMAR and only one child had bilateral visual impairment ≥1.0logMAR at final documented contact.

**Table 4:** JIA-U Ocular Outcomes per affected eyes

JIA –U					Idiopathic Uveitis/Pars Plantis			
	Current Study (*n* = 91)	Tugal-Tutkun [9] 1996 (*n* = 100)	Kadayifçilar [33] 2003 (*n* = 55)	Kump7 2005 (*n* = 165)	Current Study (n = 118)	Tugal-Tutkun^9^ 1996 (*n* = 88)	Kadayifçilar [33] 2003 (*n* = 134)	Kump^7^ 2005 (*n* = 202)
Cataract	42 (26.3%)	71 (71%)	16 (32.7%)	105 (64%)	17 (14.4%)	33 (37.5%)	41 (30.6%)	60 (29.6%)
Glaucoma	15 (9.4%)	30 (30%)	1 (2%)	33 (20%)	0	2 (2.3%)	7 (5.2%)	29 (14.4%)
Band Keratopathy	27 (16.9%)	66 (66%)	5 (10%)	76 (46%)	9 (7.6%)	16 (18.2%)	13 (9.7%)	24 (11.9%)
Post. Synechiae	40 (25%)	–	–	96 (58%)	22 (18.6%)	–	–	49 (24.3%)
Optic Disc Swelling	4 (2.5%)	6 (6%)	1 (2%)	5 (3%)	20 (16.9%)	10 (11.4%)	6 (4.5%)	14 (6.9%)
Hypotony	5 (3.1%)	19 (19%)	3 (5.5%)	17 (10%)	1 (0.8%)	2 (2.3%)	1 (0.7%)	1 (0.5%)
Macular Oedema	7 (4.4%)	–	–	–	13 (11%)	–	–	–
Vitreous Haemorrhage	1 (0.6%)	3 (3%)	0	0	0	8 (5%)	2 (1.5%)	3 (1.5%)
Epiretina Membranel	0	–	1 (2%)	17 (10%)	1 (0.8%)	–	–	20 (9.9%)

Uncertainties remain on the optimum time to initiate systemic therapy and the duration of systemic treatments for children with uveitis as well as the visual outcomes of those children with uveitis moving on to adulthood. Evidence for biologic treatments other than Adalimumab remains limited in childhood uveitis. A trial of subcutaneous tocilizumab for JIA-U is currently underway which hopefully will provide further evidence for the use of IL-6 blockade (APTITUDE) [26]. Biologic agents are expensive and carry an increased risk of infection and other side effects [27–30] which needs to be balanced against the potential benefits in reducing sight loss. We also recognise the burden to children and their families managing frequent hospital appointments, eye drops and immunosuppression regimes. Even in the absence of long term complications, uveitis can have emotional and psychological consequences for the child and family encompassing anxiety, anger and fear of the future [31].

As a retrospective study we acknowledge certain limitations to the study. The duration of follow up within the patient cohort was unequal in some cases, and as a result data is not available for all patients at all time points. Additionally, in a tertiary centre there may be overrepresentation of severe cases, and children who had mild disease may have not had been included as they are no longer managed by the service. Unfortunately therapy adverse events were not captured in this study. The era of biologic therapy has brought improvements in visual outcomes for children with uveitis but there is still potential for ongoing improvement in outcomes in the future.

Improved visual outcomes may be a result of a combination of factors. New therapies are capable of controlling inflammation refractory to conventional immunosuppressive therapy [20]. However clinical practice has evolved over the past decade to include robust and audited JIA uveitis screening standards [32], early treatment and close monitoring of affected children within a multidisciplinary team. The establishment of a combined paediatric rheumatology and uveitis clinic with specialist nurses at the Bristol Eye Hospital has been crucial in providing timely and effective monitoring and management for children with complex uveitis. The implementation of these practices may have contributed to improved outcomes.

## Conclusion

This study has demonstrated an encouraging improvement in the rate of ocular complications and visual impairment in children with non-infectious uveitis when compared to previous publications. Notably, the rate of biologic use was high (34.9%), reflecting their increasing importance in modern immunomodulation. Visual outcomes between the JIA-U and idiopathic uveitis cohorts were not significant. Cataract development was the most common ocular complication within the cohort.

## Abbreviations

CI: Confidence Interval
DMARD: Disease Modifying Antirheumatic Drug
EY: Eye Year
IOP: Intraocular Pressure
JIA: Juvenile Idiopathic Arthritis
JIA-U: Juvenile Idiopathic Arthritis associated Uveitis
mmHg: Millimeter of mercury
Pt: Patient
RCT: Randomised Control Trial
SUN: Standardization of Uveitis Nomenclature
TINU: Tubulointerstitial Nephritis and Uveitis
